# Which facets of emotion dysregulation are uniquely associated with emotional allodynia in fibromyalgia? A preliminary analysis using the emotional allodynia questionnaire and the difficulties in emotion regulation scale

**DOI:** 10.3389/fpsyg.2026.1910128

**Published:** 2026-07-29

**Authors:** Alberto Corriero, Mariateresa Giglio, Alfonso Pilolla, Filomena Galdini, Fara Fornarelli, Cinzia Di Venosa, Elisabetta Costantino, Daniela De Orsi, Giustino Varrassi, Paolo Trerotoli, Filomena Puntillo

**Affiliations:** 1 Department of Interdisciplinary Medicine – Pain and ICU Section, University of Bari Aldo Moro, Bari, Italy; 2 Nutrimente Onlus, Milano, Italy; 3 Centro di Salute Mentale Area 5, ASL Bari, Bari, Italy; 4 Paolo Procacci Foundation, Rome, Italy; 5 Department of Interdisciplinary Medicine – Statistics Section, University of Bari Aldo Moro, Bari, Italy

**Keywords:** central nervous system sensitization, DERS, emotion dysregulation, emotion regulation, emotional allodynia, fibromyalgia

## Abstract

**Background:**

Emotional allodynia, a condition in which neutral or low-intensity interpersonal stimuli provoke disproportionate emotional distress, has been proposed as a clinically relevant feature of fibromyalgia. The Emotional Allodynia Questionnaire (AEQ) was recently preliminarily validated and showed a strong association with emotion dysregulation as measured by the Difficulties in Emotion Regulation Scale (DERS). However, which specific DERS facets underlie emotional allodynia, independently of depression, anxiety, pain catastrophizing and central sensitization, remains unknown.

**Methods:**

Cross-sectional data from 136 adults with fibromyalgia (91.9% female; mean age 57.6 ± 11.3 years) were analyzed. AEQ total score was examined against each DERS subscale (Non-Acceptance, Goals, Impulse, Awareness, Strategies, Clarity) using Spearman correlations, partial correlations controlling for BDI-II, STAI-Y2, PCS and CSI, and hierarchical linear regression with incremental R^2^.

**Results:**

At zero order, five subscales correlated with AEQ with the exception of Awareness. After controlling for all covariates, Impulse (ρ_partial = 0.286, *p* = 0.001), Strategies (ρ_partial = 0.245, *p* = 0.005), Non-Acceptance (ρ_partial = 0.220, *p* = 0.011) and Clarity (ρ_partial = 0.201, *p* = 0.021) remained significantly associated with AEQ, and each contributed incremental variance above the base model (ΔR^2^ = 0.019–0.038, *p* < 0.05). Awareness was not significant.

**Conclusion:**

Emotional allodynia in fibromyalgia is associated with loss of behavioral control (Impulse), limited confidence in emotion regulation (Strategies), non-acceptance and difficulty identifying emotional states (Clarity), but not reduced attention to emotional states (Awareness), suggesting emotions that are noticed but difficult to identify and regulate, with implications for targeted psychological treatments.

## 1 Introduction

Fibromyalgia is a chronic widespread pain condition with somatic hyperalgesia, allodynia, and a high rate of psychological comorbidity (Raja et al., 2020). It is now classified as a nociplastic condition, where central sensitization amplifies both nociceptive and affective signals (Kosek et al., 2016). Beyond somatic hypersensitivity, patients with fibromyalgia show elevated negative emotionality and heightened emotional reactivity to everyday stressors, responding with an intensity that appears disproportionate to the eliciting situation (Van Middendorp et al., 2008; Renz et al., 2025). Integrative models propose that in fibromyalgia the brain's threat-detection circuits are persistently over-engaged while the systems that normally down-regulate distress are under-active, which keeps the salience (midcingulo-insular) network in a continuous state of alert (Pinto et al., 2023). In this state even minor emotional cues can elicit a disproportionate response, so a lowered threshold for affective activation is an expected feature of the condition. Such affective-cognitive amplification itself may be one of several mechanisms that contribute to nociplastic pain, alongside central sensitization and altered descending modulation (Kosek et al., 2021).

We have proposed the term emotional allodynia to describe this pattern: by analogy with somatic allodynia, neutral or low-intensity interpersonal stimuli provoke disproportionate emotional distress (Corriero et al., 2026). To measure this, we developed the Emotional Allodynia Questionnaire (AEQ), an 11-item self-report scale preliminarily validated in 107 fibromyalgia patients (Corriero et al., 2026). In that study, the correlation with global emotion dysregulation [Difficulties in Emotion Regulation Scale (DERS) total; Spearman ρ = 0.61] was among the strongest associations observed. Conceptually, emotional allodynia denotes a lowered threshold of emotional activation, whereas emotion dysregulation concerns the difficulty of managing emotional responses once activated (Gratz and Roemer, 2004); a patient may show a low activation threshold while retaining adequate regulatory capacity, and the reverse can also occur. The construct is related to but distinct from rejection sensitivity, the anxious expectation of rejection (Downey and Feldman, 1996), interpersonal sensitivity as a stable trait (Boyce and Parker, 1989), and affective instability, which refers to broad mood swings not tied to interpersonal triggers. In each case the contrast is the same: emotional allodynia is defined by the magnitude of the affective reaction to an actual low-intensity interpersonal cue, not by an anticipatory expectation of rejection (rejection sensitivity), a broad and stable personality disposition (interpersonal sensitivity), or context-free mood fluctuations (affective instability).

The DERS yields six subscales: non-acceptance of negative emotions (Non-Acceptance), difficulty functioning when distressed (Goals), impulse control problems when upset (Impulse), limited regulation strategies (Strategies), poor attention to emotional states (Awareness), and difficulty identifying emotions (Clarity). These facets do not map onto the same clinical targets.

Knowing that emotional allodynia and dysregulation correlate globally is a starting point, not an answer. If Impulse is the relevant mechanism, Dialectical Behavior Therapy (DBT)-based distress tolerance work seems appropriate (Linehan, 1993). If Clarity is central, the picture resembles alexithymia, a difficulty in identifying and describing one's own emotional states (Bagby et al., 1994; Gratz and Roemer, 2004), and calls for different approaches. Prior studies in fibromyalgia have linked DERS subscales to pain-related distress and disability, consistently identifying Non-Acceptance of emotional responses as the most prominent facet (Trucharte et al., 2020; Frumer et al., 2023). However, these studies did not control for comorbid depression and anxiety, leaving open the question of which subscales retain unique associations beyond shared psychopathological variance. Whether emotion dysregulation facets show the same pattern when examined against emotional allodynia, rather than against general distress or disability, is an open question.

Given that nearly half the sample has comorbid depression, and that depression, anxiety, and emotion dysregulation overlap considerably, it remains unclear whether associations between DERS subscales and AEQ reflect a specific emotion-allodynia mechanism or are largely explained by shared psychopathological variance. Disentangling these contributions was the primary aim of this study.

This study examines which DERS facets are uniquely associated with AEQ using data from the ongoing PEARL study, controlling for the potential confounding role of comorbid depression, anxiety, pain catastrophizing, and central sensitization.

## 2 Methods

### 2.1 Participants and procedure

Data were drawn from the PEARL study, an ongoing cross-sectional psychometric study conducted at the Pain Clinic of Policlinico Hospital, University of Bari Aldo Moro, Bari, Italy. Fibromyalgia was diagnosed according to American College of Rheumatology (ACR) 2016 criteria (Wolfe et al., 2016). All participants provided written informed consent prior to enrolment, and the study was approved by the Local Ethics Committee (approval number: 2451/CEL; see Ethics Statement).

After clinical screening, eligible participants completed a battery of self-report questionnaires in paper format during their clinical visit. A physician was available throughout to clarify any items or instructions upon request. Completed questionnaires were subsequently verified and entered into a secure digital platform. The estimated completion time was approximately 25–30 min. The analysis sample comprised *N* = 136 adults with fibromyalgia (125 female, 11 male; mean age 57.6 ± 11.3 years, range 33–85 years). This sample represents an updated cohort from the ongoing PEARL study, extending the preliminary validation dataset reported in Corriero et al. (2026). No a priori power analysis was performed; the sample comprised all eligible PEARL participants available at the time of analysis. The physician-verified paper procedure left no item-level missing data, and analyses used complete cases.

### 2.2 Measures

**Emotional allodynia questionnaire (AEQ):** 11-item self-report scale assessing the tendency to experience disproportionate emotional distress in response to neutral or low-intensity interpersonal stimuli, rated on a 5-point Likert scale (0–4; total range 0–44); higher scores indicate greater emotional allodynia. AEQ served as the dependent variable (Corriero et al., 2026).

**Difficulties in emotion regulation scale, DERS** (Gratz and Roemer, 2004): 33-item [Italian adaptation, Sighinolfi et al. (2010)] instrument yielding six subscale scores [Non-Acceptance (6 items), Goals (5 items), Impulse (6 items), Strategies (8 items), Clarity (5 items), and Awareness (3 items)]; higher scores indicate greater difficulties. Each subscale served as an independent variable.

**Beck depression inventory-II, BDI-II** (Beck et al., 2011): measures depressive symptom severity. The BDI-II, STAI-Y2, PCS, and CSI served as covariates in all analyses.

**State-trait anxiety inventory, trait subscale, STAI-Y2** (Spielberger, 2012): measures trait anxiety.

**Pain catastrophizing scale, PCS** (Sullivan et al., 1995): measures pain-related catastrophic thinking.

**Central sensitization inventory, CSI** (Mayer et al., 2012): measures symptoms associated with central sensitization.

### 2.3 Statistical analysis

Associations between AEQ and each DERS subscale were first examined using Spearman rank-order correlations (zero-order). To isolate unique associations independent of depression, anxiety, pain catastrophizing, and central sensitization, partial Spearman correlations were computed controlling simultaneously for BDI-II, STAI-Y2, PCS, and CSI using the ppcor package in R (Kim, 2015).

Hierarchical linear regression was used to quantify incremental explained variance. Step 1 included the four covariates; in Step 2, each DERS subscale was added individually (not simultaneously, to avoid suppression effects due to inter-subscale collinearity), and the F-change statistic was computed. Given the exploratory nature of the study, no correction for multiple comparisons was applied; alpha was set at 0.05 (two-tailed). All analyses were conducted in R version 4.5.1 (R Core Team, 2025).

## 3 Results

### 3.1 Sample characteristics

Sociodemographic, clinical, and treatment characteristics of the sample are presented in Supplementary Table S1. The sample was predominantly female (91.9%), with a mean age of 57.6 years (SD = 11.3). The majority of participants had been living with pain for more than 10 years (52.2%), reported sedentary physical activity levels (60.3%), and scored in the moderate-to-severe range on depression (BDI-II M = 22.12, SD = 10.24) and trait anxiety (STAI-Y2 M = 47.73, SD = 9.26) measures. AEQ total scores ranged from 1 to 41 (*M* = 17.85, SD = 9.95; Cronbach's α = 0.905, 95% CI: 0.880–0.927), with no floor or ceiling effects. Internal consistency for all measures in the present sample is reported in Supplementary Table S2.

### 3.2 DERS subscale descriptives

Descriptive statistics for DERS subscales are presented in Table 1.

**Table 1:** DERS subscale descriptives (*n* = 136).

DERS subscale	Items (*n*)	*M*	SD	Range
Non-Acceptance	6	13.9	6.3	6–30
Goals	5	14.9	5.1	5–25
Strategies	8	18.8	7.4	8–38
Impulse	6	12.0	4.9	6–30
Clarity	5	11.1	4.4	5–25
Awareness	3	7.0	3.5	3–15
**Total DERS**	**33**	**77.49**	**22.33**	**37–150**

M, mean; SD, standard deviation.

### 3.3 Zero-order Spearman correlations

Five of six DERS subscales were significantly correlated with AEQ (Table 2). Impulse (ρ = 0.575, *p* < 0.001) and Strategies (ρ = 0.570, *p* < 0.001) showed the strongest zero-order associations, followed by Non-Acceptance (ρ = 0.452, *p* < 0.001), Goals (ρ = 0.425, *p* < 0.001), and Clarity (ρ = 0.414, *p* < 0.001). Awareness was not significantly associated with AEQ (ρ = 0.048, *p* = 0.576).

**Table 2:** Spearman and partial correlations: AEQ × DERS subscales.

DERS subscale	Zero-order		Partial	
	ρ	*p*	ρ	*p*
Impulse	0.575	< 0.001^***^	0.286	0.001^***^
Strategies	0.570	< 0.001^***^	0.245	0.005^**^
Non-Acceptance	0.452	< 0.001^***^	0.220	0.011^*^
Clarity	0.414	< 0.001^***^	0.201	0.021^*^
Goals	0.425	< 0.001^***^	0.153	0.079
Awareness	0.048	0.576	−0.018	0.833

Partial correlations control simultaneously for BDI-II, STAI-Y2, PCS, and CSI. ^*^p < 0.05; ^**^p < 0.01; ^***^p < 0.001.

### 3.4 Partial correlations

**Figure 1:**
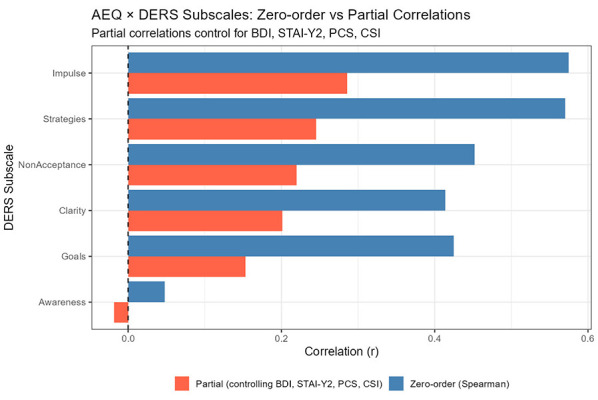
Zero-order Spearman correlations (blue bars) and partial correlations (red bars) between AEQ total score and each DERS subscale (*n* = 136). Subscales are ordered by partial ρ magnitude. Zero-order correlations reflect the raw association between AEQ and each subscale without any adjustment. Partial correlations reflect the unique association remaining after simultaneously controlling for depression (BDI-II), trait anxiety (STAI-Y2), pain catastrophizing (PCS), and central sensitization (CSI). A large reduction from blue to red bar indicates that the association was largely accounted for by the covariates; a small reduction indicates that the subscale retains a specific, independent relationship with emotional allodynia. Dashed line at ρ = 0.

After controlling for BDI-II, STAI-Y2, PCS, and CSI, four subscales retained significant unique associations with AEQ (Table 2, Figure 1): Impulse (ρ_partial = 0.286, *p* = 0.001), Strategies (ρ_partial = 0.245, *p* = 0.005), Non-Acceptance (ρ_partial = 0.220, *p* = 0.011), and Clarity (ρ_partial = 0.201, *p* = 0.021). Goals did not reach significance (ρ_partial = 0.153, *p* = 0.079). Awareness was not significant (ρ_partial = −0.018, *p* = 0.833). Ninety-five percent confidence intervals for all zero-order and partial correlations are provided in Supplementary Table S3.

### 3.5 Hierarchical regression: incremental R^2^

The base model (BDI-II + STAI-Y2 + PCS + CSI) explained *R*^2^ = 0.421 of variance in AEQ. The addition of each DERS subscale individually produced significant incremental variance for Impulse (ΔR^2^ = 0.038, *p* = 0.003), Strategies (ΔR^2^ = 0.028, *p* = 0.011), Clarity (ΔR^2^ = 0.028, *p* = 0.011), Non-Acceptance (ΔR^2^ = 0.019, *p* = 0.039), and Goals (ΔR^2^ = 0.018, *p* = 0.042). Awareness (ΔR^2^ =0.000, *p* = 0.746) did not significantly increment prediction. Results are summarized in Table 3. When all six DERS subscales were entered simultaneously together with the four covariates, the block accounted for significant additional variance overall (ΔR^2^ = 0.071, *p* = 0.011), but no individual subscale remained independently significant, consistent with substantial shared variance among the correlated facets. Full model estimates, collinearity, and regression diagnostics are reported in Supplementary Tables S4, S5.

**Table 3:** Incremental *R*^2^ for each DERS subscale added to base model.

DERS subscale	*R^2^* (model)	ΔR^2^	*p* (F-change)
Impulse	0.459	0.038	0.003^**^
Strategies	0.449	0.028	0.011^*^
Clarity	0.449	0.028	0.011^*^
Non-Acceptance	0.440	0.019	0.039^*^
Goals	0.439	0.018	0.042^*^
Awareness	0.421	0.000	0.746

Base model *R*^2^ = 0.421 (BDI-II + STAI-Y2 + PCS + CSI). Each subscale was added individually to the base model. ^*^p < 0.05, ^**^p < 0.01.

## 4 Discussion

The aim of this study was to identify which specific facets of emotion dysregulation are uniquely associated with emotional allodynia in fibromyalgia, beyond the variance explained by depression, anxiety, pain catastrophizing, and central sensitization. Across three analytic approaches, a consistent and interpretable pattern emerged.

Partial correlations were substantially attenuated relative to zero-order values (approximately 50% reduction on average), reflecting expected overlap with depression, anxiety, and catastrophizing. Non-etheless, four subscales retained significant unique associations (ρ_partial = 0.20–0.29, all *p* < 0.05), indicating that the link between emotional allodynia and emotion dysregulation is not reducible to comorbid psychopathology, supporting the discriminant validity of AEQ as a construct distinct from general emotional distress. Furthermore, these findings move beyond the global AEQ-DERS association reported in the preliminary validation study [Spearman ρ = 0.61; Corriero et al. (2026)]: not all facets of dysregulation are equally relevant to emotional allodynia, and a specific profile emerges: emotional allodynia is associated with the reactive and regulatory facets of dysregulation (Impulse, Strategies, Non-Acceptance, and Clarity) but not with reduced attentional engagement with emotional states (Awareness).

The significant unique association of Non-Acceptance with AEQ is consistent with its prominent role in prior DERS research in fibromyalgia (Trucharte et al., 2020; Frumer et al., 2023), suggesting that Non-Acceptance of emotional responses may be relevant across different emotional outcomes in this population. The additional associations with Impulse and Clarity observed here extend prior findings by identifying facets that may be specifically relevant to emotional reactivity, an outcome not previously examined in this literature.

Impulse, Strategies, Non-Acceptance, and Clarity showed significant unique associations with AEQ across both partial correlations and incremental regression, with Impulse and Strategies the most robust across all analytic approaches. Goals did not reach significance in partial correlations (ρ_partial = 0.153, *p* = 0.079), though it retained a marginal incremental contribution in the regression analysis (Δ*R*^2^ = 0.018, *p* = 0.042), suggesting a weak and largely shared association. Most strikingly, Awareness showed no relationship with AEQ at any level of analysis.

The association of AEQ with Strategies is conceptually consistent with the construct of emotional allodynia. In the Italian DERS adaptation (Sighinolfi et al., 2010), the Strategies subscale captures pessimism about one's capacity to regulate distress: patients believe they will remain upset for a long time, that nothing can be done to feel better, and that rumination is the only available response. Patients scoring high on AEQ also tended to score higher on Strategies, suggesting that disproportionate emotional responsiveness may co-occur with a sense of helplessness about emotional regulation, though causal inference is not possible from cross-sectional data.

The association with Impulse also warrants consideration. The DERS Impulse subscale captures loss of behavioral control and a sense of emotional overwhelm when distressed; higher scores reflect difficulty maintaining behavioral regulation and a perception that emotions are beyond one's control. The positive association with AEQ suggests that patients high in emotional allodynia tend to experience greater loss of control when emotionally activated. Prior studies in fibromyalgia have examined Impulse-related facets in the context of general distress and disability (Kökönyei et al., 2014; Trucharte et al., 2020). The significant association with AEQ observed here suggests that loss of emotional control may be specifically relevant to emotional reactivity rather than to fibromyalgia-related distress or disability more broadly.

Potentially the most theoretically informative finding is the dissociation between Clarity and Awareness. The Clarity subscale, capturing difficulty identifying and labeling emotional states (Gratz and Roemer, 2004), a pattern that overlaps with, but is not equivalent to, alexithymia, showed a significant unique association with AEQ. In contrast, the Awareness subscale, capturing failure to pay attention to emotional states, showed no association. This dissociation suggests that emotional allodynia is associated with difficulty identifying and making sense of emotional states rather than with reduced attention to them. This profile is distinct from classical alexithymia, in which both reduced awareness and difficulty identifying emotions co-occur (Castelli et al., 2012). Replication using validated alexithymia measures (e.g., TAS-20) is needed to confirm this distinction. The null Awareness association should also be read in light of documented psychometric concerns about this subscale, which loads weakly on the general DERS factor and is sometimes excluded from the total score (Mancinelli et al., 2024), consistent with Awareness reflecting emotional attention rather than dysregulation as such.

These findings may carry preliminary therapeutic implications, though replication in clinical trials is needed before any treatment recommendations can be made. The pattern of significant associations with Impulse, Strategies, Non-Acceptance, and Clarity, alongside a non-significant association with Awareness, may suggest a role for interventions targeting loss of emotional control (DBT-based distress tolerance), acceptance of negative emotions, limited regulation strategies, and difficulty identifying emotions [e.g., emotion-focused approaches, Linehan (1993)]. The significant association with Clarity and the non-significant Awareness suggest that approaches combining emotion regulation training with emotion identification work may be particularly relevant. These are preliminary hypotheses that require prospective and experimental verification. Consistent with this, non-pharmacological interventions addressing emotional and interoceptive processing, such as amygdala and insula retraining or physical activity, have shown benefit over standard medication in fibromyalgia (Norouzi et al., 2025).

One might argue that the DERS Impulse subscale alone could substitute for AEQ in clinical settings, given the overlap between the two measures. However, AEQ and DERS Impulse share only about 33% of variance (ρ = 0.575), and the two scales operationalise conceptually distinct constructs. DERS Impulse measures loss of behavioral control when emotionally overwhelmed, applicable across populations and contexts. AEQ was specifically designed to capture the tendency to experience disproportionate emotional distress in response to neutral or low-intensity interpersonal stimuli (Corriero et al., 2026), operationalising emotional allodynia within a nociplastic pain framework. Moreover, AEQ (11 items) is considerably more parsimonious than the full DERS (33 items in the italian version) and was normed in a chronic pain population. Studies examining the incremental validity of AEQ over DERS Impulse in predicting pain-specific outcomes are warranted to further clarify this distinction.

This study should be regarded as a preliminary, exploratory analysis rather than a formal interim analysis. It was prompted by the encouraging associations that have emerged as the ongoing PEARL study has progressed, and it addresses a focused secondary question within a continuing data collection; it was not derived from a pre-specified interim analysis plan, and its findings are therefore offered as hypothesis-generating.

The study has several limitations. The sample size (*n* = 136) was sufficient for the planned analyses but limits the precision of partial correlation estimates and the generalizability of findings. The cross-sectional design precludes causal inference. The AEQ is a recently validated instrument and replication of these subscale findings in independent samples is needed. Effect sizes for partial correlations were small (ρ_partial = 0.20–0.29), and the incremental variance explained by individual subscales was modest (ΔR^2^ = 2–4%), reflecting the high overlap between covariates and DERS subscales. Given the exploratory framing of the study, no correction for multiple comparisons was applied, and findings should be interpreted accordingly. Because all measures were self-reported within a single session, the associations may also reflect common method variance and generalized negative affectivity.

Finally, the single tertiary-center setting and the predominantly female sample limit generalizability to other settings and to male patients.

## 5 Conclusion

In fibromyalgia, emotional allodynia shows specific and consistent associations with loss of emotional and behavioral control (Impulse), limited confidence in emotion regulation (Strategies), Non-Acceptance of negative emotions, and difficulty identifying emotional states (Clarity), independent of comorbid depression, anxiety, pain catastrophizing, and central sensitization. The absence of an association with Awareness suggests that emotional allodynia does not involve reduced attention to emotions, but rather difficulty identifying and regulating them. Emotions are perceived but difficult to process. If replicated, these findings could inform the design of targeted psychological treatments for fibromyalgia patients with high emotional burden.

## Data Availability

The raw data supporting the conclusions of this article will be made available by the authors, without undue reservation.
