# Supplementary material for: Which facets of emotion dysregulation are uniquely associated with emotional allodynia in fibromyalgia? A preliminary analysis using the emotional allodynia questionnaire and the difficulties in emotion regulation scale
Source: Front Psychol. 2026 Jul 29;17:1910128. doi: 10.3389/fpsyg.2026.1910128 (PMC13462364; doi:10.3389/fpsyg.2026.1910128)
Supplement: Supplementary file 1 [file Supplementary_file_1.docx]

**Supplementary Material**

Which Facets of Emotion Dysregulation Are Uniquely Associated With Emotional Allodynia in Fibromyalgia? A Preliminary Analysis Using the Emotional Allodynia Questionnaire and the Difficulties in Emotion Regulation Scale

**Supplementary Table S1. Sociodemographic, clinical, and treatment characteristics of the study sample (N = 136)**

| **Variable** | **Overall (N = 136)** |
| --- | --- |
| **Sample and demographics** |  |
| n | 136 |
| Age, mean (SD) | 57.62 (11.26) |
| Male sex, n (%) | 11 (8.1) |
| Occupation, n (%) |  |
| Unemployed / homemaker | 71 (52.2) |
| Employed | 42 (30.9) |
| Retired | 23 (16.9) |
| Level of education, n (%) |  |
| Primary school | 11 (8.1) |
| Middle school | 55 (40.4) |
| High school | 54 (39.7) |
| University degree | 16 (11.8) |
| Marital status, n (%) |  |
| Married | 96 (70.6) |
| Single | 12 (8.8) |
| Divorced / Separated / Widowed | 28 (20.6) |
| Live alone, n (%) | 22 (16.2) |
| **Pain characteristics** |  |
| Pain duration, n (%) |  |
| Between 6 months and 1 year | 4 (2.9) |
| Between 1 and 3 years | 15 (11.0) |
| More than 3 years | 20 (14.7) |
| More than 5 years | 26 (19.1) |
| More than 10 years | 71 (52.2) |
| VAS pain, median (IQR) | 8 (7–10) |
| **Lifestyle and self-rated outcomes** |  |
| Physical activity level, n (%) |  |
| Sedentary (less than 1 hour/week) | 82 (60.3) |
| Light (1 to 2 hours/week) | 34 (25.0) |
| Moderate (3 to 5 hours/week) | 18 (13.2) |
| Vigorous (6 hours/week or more) | 2 (1.5) |
| Subjective sleep quality (0 to 10), mean (SD) | 4.44 (2.62) |
| Subjective quality of life (0 to 10), mean (SD) | 4.57 (2.15) |
| **Psychosocial and relationship history** |  |
| Current romantic relationship, n (%) | 106 (77.9) |
| Relationship duration > 3 years, n (%)ᵃ | 102 (96.2) |
| Time since last relationship, n (%)ᵇ |  |
| Between 6 months and 1 year | 1 (3.3) |
| Between 1 and 3 years | 1 (3.3) |
| More than 3 years | 28 (93.3) |
| Significant breakup in last 3 years, n (%) | 20 (14.7) |
| Still emotionally involved, n (%)ᶜ | 14 (70.0) |
| Social support available, n (%) | 109 (80.1) |
| Emotional support level (0 to 10), mean (SD) | 5.87 (3.26) |
| Abandonment/betrayal/rejection history, n (%) |  |
| Never | 55 (40.4) |
| Once | 32 (23.5) |
| Multiple times | 49 (36.0) |
| Distress from perceived distance, n (%) | 106 (77.9) |
| Rapid intense attachment tendency, n (%) | 56 (41.2) |
| Current psychotherapy, n (%) | 20 (14.7) |
| **Medication history, pain-related** |  |
| SNRI use, n (%) | 22 (16.2) |
| SSRI use, n (%) | 20 (14.7) |
| Opioid use, n (%) | 35 (25.7) |
| Medical cannabis use, n (%) | 17 (12.5) |
| NSAID use, n (%) | 28 (20.6) |
| Corticosteroid use, n (%) | 13 (9.6) |
| Paracetamol use, n (%) | 28 (20.6) |
| Muscle relaxant use, n (%) | 29 (21.3) |
| Benzodiazepine use, n (%) | 39 (28.7) |
| Gabapentinoid use, n (%) | 51 (37.5) |

*Note. SD = standard deviation; IQR = interquartile range; VAS = Visual Analogue Scale; SNRI = serotonin-norepinephrine reuptake inhibitor; SSRI = selective serotonin reuptake inhibitor; NSAID = non-steroidal anti-inflammatory drug. ᵃ Among participants currently in a relationship (n = 106). ᵇ Among participants not currently in a relationship (n = 30). ᶜ Among participants who reported a significant breakup in the last 3 years (n = 20). Medication use coded from free-text responses (current therapies and specific psychotropic fields) using standardized keyword dictionaries; values may be underestimates.*

**Supplementary Table S2. Internal consistency of all measures in the present sample (n = 136)**

*Note. k = number of items. α = Cronbach's alpha; ω = McDonald's omega.*

| **Measure** | **k** | **Cronbach's α** | **McDonald's ω** |
| --- | --- | --- | --- |
| AEQ | 11 | 0.905 | 0.906 |
| PCS | 13 | 0.898 | 0.905 |
| CSI | 25 | 0.857 | 0.865 |
| BDI-II | 21 | 0.873 | 0.884 |
| STAI-Y2 | 20 | 0.909 | 0.912 |
| DERS total | 33 | 0.902 | 0.909 |
| DERS Non-Acceptance | 6 | 0.829 | 0.836 |
| DERS Strategies | 8 | 0.818 | 0.823 |
| DERS Impulse | 6 | 0.759 | 0.787 |
| DERS Awareness | 3 | 0.750 | 0.762 |
| DERS Goals | 5 | 0.744 | 0.757 |
| DERS Clarity | 5 | 0.668 | 0.677 |

**Supplementary Table S3. Zero-order and partial correlations between AEQ and DERS subscales, with 95% confidence intervals (n = 136)**

*Note. Partial correlations control simultaneously for BDI-II, STAI-Y2, PCS, and CSI. Confidence intervals computed via Fisher's z transformation.*

| **DERS subscale** | **Zero-order ρ [95% CI]** | **Partial ρ [95% CI]** |
| --- | --- | --- |
| Impulse | 0.575 [0.44, 0.69] | 0.286 [0.12, 0.44] |
| Strategies | 0.570 [0.43, 0.68] | 0.245 [0.08, 0.40] |
| Non-Acceptance | 0.452 [0.30, 0.58] | 0.220 [0.05, 0.38] |
| Clarity | 0.414 [0.26, 0.55] | 0.201 [0.03, 0.36] |
| Goals | 0.425 [0.27, 0.56] | 0.153 [-0.02, 0.32] |
| Awareness | 0.048 [-0.12, 0.22] | -0.018 [-0.19, 0.15] |

**Supplementary Table S4. Multiple regression of AEQ on all six DERS subscales entered simultaneously with the four covariates (n = 136)**

*Note. Outcome: AEQ total. Base model (BDI-II + STAI-Y2 + PCS + CSI) R² = .421; full model R² = .492; ΔR² = .071, F(6,125) = 2.91, p = .011. β = standardized coefficient; VIF = variance inflation factor.*

| **Predictor** | **b [95% CI]** | **β** | **p** | **VIF** |
| --- | --- | --- | --- | --- |
| BDI-II | -0.094 [-0.31, 0.12] | -0.096 | .388 | 3.05 |
| STAI-Y2 | 0.213 [-0.01, 0.43] | 0.198 | .060 | 2.68 |
| PCS | 0.211 [0.06, 0.36] | 0.249 | .006 | 1.94 |
| CSI | 0.043 [-0.06, 0.15] | 0.069 | .409 | 1.68 |
| Non-Acceptance | 0.125 [-0.11, 0.37] | 0.079 | .303 | 1.45 |
| Goals | 0.084 [-0.26, 0.43] | 0.043 | .634 | 2.00 |
| Clarity | 0.339 [-0.02, 0.70] | 0.151 | .065 | 1.62 |
| Strategies | 0.181 [-0.11, 0.47] | 0.135 | .218 | 2.92 |
| Awareness | -0.195 [-0.60, 0.21] | -0.070 | .341 | 1.30 |
| Impulse | 0.236 [-0.15, 0.63] | 0.115 | .232 | 2.27 |

**Supplementary Table S5. Regression diagnostics for the hierarchical model (n = 136)**

*Note. Diagnostics computed on the full model (AEQ ~ four covariates + six DERS subscales).*

| **Diagnostic** | **Result** |
| --- | --- |
| Residual normality (Shapiro-Wilk) | W = 0.993, p = .74 |
| Homoscedasticity (Breusch-Pagan) | χ² = 6.71, df = 10, p = .75 |
| Influential observations (Cook's distance) | max = 0.10 (none > 1) |
| Multicollinearity (VIF) | max = 3.05, mean = 2.09 |
